# Multiple Sclerosis and SARS-CoV-2 Vaccination: Considerations for Immune-Depleting Therapies

**DOI:** 10.3390/vaccines9020099

**Published:** 2021-01-28

**Authors:** Johann Sellner, Paulus S. Rommer

**Affiliations:** 1 Department of Neurology, Landesklinikum Mistelbach-Gänserndorf, 2130 Mistelbach, Austria; 2 Department of Neurology, Klinikum rechts der Isar, Technische Universität München, 81675 München, Germany; 3 Department of Neurology, Christian Doppler Medical Center, Paracelsus Medical University, 5020 Salzburg, Austria; 4 Department of Neurology, Medical University of Vienna, 1090 Vienna, Austria; paulus.rommer@meduniwien.ac.at

**Keywords:** vaccination, COVID-19, SARS-CoV-2, multiple sclerosis, disease-modifying drugs, immune-depleting therapies, CD20 depletion, cladribine, alemtuzumab

## Abstract

Several concerns have been raised about the use of immunodepleting agents including alemtuzumab, cladribine and CD20-depleting antibodies in people with multiple sclerosis (pwMS) during the coronavirus disease (COVID) 2019 pandemic. As the end of the pandemic is not yet in sight, vaccination against severe acute respiratory syndrome coronavirus type 2 (SARS-CoV-2) may be an elegant strategy to overcome the potential hazards associated with initiating and continuing treatment with immune-depleting agents. In this review, we summarize the immunological effects of immune-depleting therapy and underlying considerations for the hitherto existing recommendations that suggest a restricted use of immune-deleting therapies during the pandemic. Moreover, we critically discuss open questions regarding vaccination in general and against SARS-CoV-2 in pwMS.

## 1. Introduction

The emergence of novel coronavirus infectious disease 2019 (COVID-19), a complex clinical syndrome caused by severe acute respiratory syndrome coronavirus type-2 (SARS-CoV-2), poses an ongoing challenge for neurologists caring for people with multiple sclerosis (pwMS) [1]. Uncertainty and anxiety are frequently expressed by the patients and caregivers and are underpinned by the question of whether MS as an independent comorbidity and the use of immunotherapies are associated with an increased risk of severe COVID-19 infection [2,3]. The considerations are justified because inflammation is an integral part of disease pathogenesis, infections can exacerbate MS-related disease activity, and pwMS are at risk of life-threatening infections [4].

Using a general population risk stratification algorithm for COVID-19 related mortality, the proportion of pwMS with apparent higher risk in a population-based cohort was calculated [5]. Moderate risk was estimated for 8.8% and high/very high risk for 0.9%. Concerns about additional MS-related factors that may contribute to unfavorable outcome are gradually being confirmed by case studies, small cohorts and registries. Age, obesity and higher level of disability were independent risk factors for severe COVID-19 in a large registry-based cohort study [6]. Another study reported that pwMS with a more severe COVID-19 course were not only older and had a higher degree of disability but were also more likely to suffer from a progressive phenotype [3].

**Figure 1:**
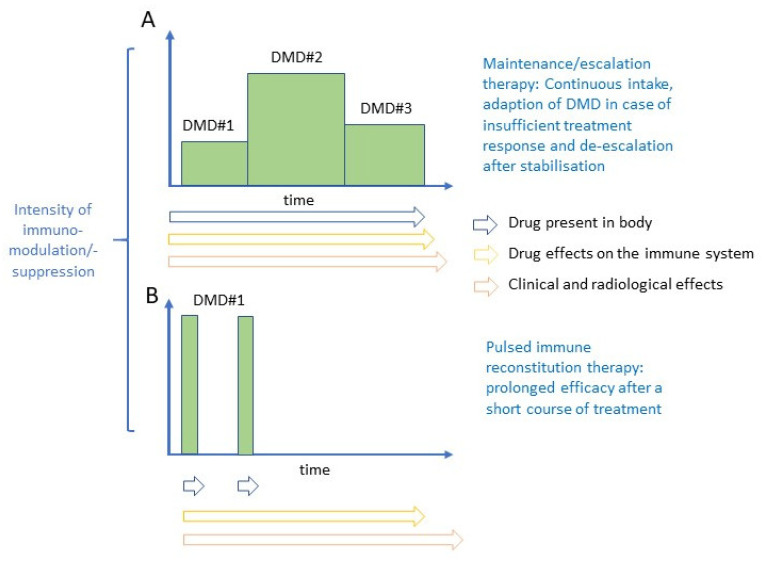
Principles of maintenance/escalation therapy (**A**) and immune-depleting therapy (**B**).

Disease-modifying drugs (DMDs) used for maintenance/escalation therapy do not exert their beneficial effect after cessation of therapy. However, some new highly effective DMDs show prolonged treatment effects after a brief treatment course. Such therapies have been termed pulsed immunodepleting, lymphodepleting or immune reconstitution therapies. The underlying concepts of maintenance/escalation therapy and pulsed immune reconstitution therapy are illustrated in Figure 1.

Typical representatives of maintenance therapies are interferon-β formulations, glatiramer acetate, S1P-receptor modulators, natalizumab and teriflunomide. Immune-depleting therapies approved for the treatment of MS are alemtuzumab, cladribine and ocrelizumab. The key features of use and depletion are summarized in Table 1.

## 2. Immunological Consequences of Immune-Depleting Therapies Approved for the Treatment of MS and Infectious Complications

### 2.1. Alemtuzumab

Alemtuzumab (Lemtrada^®^, Sanofi, Belgium) is a humanized monoclonal IgG1-antibody that targets cluster of differentiation 52 (CD52). This surface molecule with, for the most part, unknown functions is predominantly expressed on B and T cells [8,9]. Monocytes, macrophages, and eosinophils have lower expression levels of CD52. Mature NK cells, plasma cells, neutrophils, and hematological stem cells have little or no CD52 expression [10].

The approval of alemtuzumab for the treatment of active MS by the European Medicines Agency (EMA) in 2013 was based on two pivotal randomized Phase III trials. These open-label rater-blinded studies compared treatment with alemtuzumab to (subcutaneous (SC) interferon-β-1a (IFN-β, Rebif^®^, Merck, The Netherlands) in patients with relapsing-remitting MS (RRMS). The study cohort consisted of patients that were either naïve to DMD therapy (CARE-MS I) or who had relapsed while on prior DMD (CARE-MS II).

One full course of alemtuzumab consists of 12 mg daily and administered intravenously (IV) for five consecutive days and repeated after one year for three consecutive days [11]. Alemtuzumab leads to a rapid and long-lasting depletion of CD52-positive cells by two different modes of action. These include antibody-dependent, cell-mediated cytolysis (ADCC) and complement dependent cytolysis (CDC) [12]. The depletion is succeeded by a slow repopulation that arises from hematopoietic precursor cells [8]. Both, quantitative and qualitative changes in the immune-cell repertoire are observed. It has been speculated that these could initiate a reset of adaptive immune responses and subsequent long-standing efficacy. The exact mechanisms contributing to the reprogramming of the immune system has not been deciphered so far. Yet, there a specific pattern of immune-cell repopulation in peripheral blood [13]. B cells are rapidly depleted and not only return to reference levels within six months, but also show an overshoot to 124–165% of baseline levels at 12 months [14]. The CD8+ T cell count is restored after 31 months, whereas CD4+ T cells need around 60 months for complete reconstitution. The rapid CD19+ B cell subset repopulation in the absence of T cell recovery may play a decisive role for adverse effects, e.g., secondary autoimmunity [15]. Hemolytic anemia, idiopathic thrombocytopenic purpura and Grave’s disease are indeed B-cell mediated autoimmune disorders. Both naïve and memory CD4+ T cells are substantially depleted [15]. At follow-up of six years from alemtuzumab therapy, naïve and memory CD4+ and CD8+ T cells had slow repopulation kinetics and remained below reference values [16]. Notably, treatment regimen and dose-free periods were not specified in this analysis. The decrease in central memory T cells is of interest, since TH17 cells are defined by this T cell phenotype and contribute to the pathogenesis of MS [17]. Monocytes reach baseline levels after three months. Furthermore, the depletion of natural killer (NK) cells (CD16+ and CD56+) is less marked. An expansion in CD56^bright^ NK cells is observed [18]. Immunoregulatory properties have been ascribed to NK cells and the impact on disease progression is currently being evaluated [19].

Infections related to alemtuzumab are mostly mild to moderate. The spectrum includes oral herpes, herpes zoster, upper respiratory-tract infection, urinary-tract infection, influenza, and localized superficial fungal infections [20,21,22]. Since Herpes-virus infections were increased in clinical trials of alemtuzumab, prophylactic treatment with an oral anti-herpes drug from the first day of alemtuzumab infusion and for the duration of one month is mandatory [23]. Tuberculosis has been reported in patients treated with alemtuzumab; thus, before the initiation of therapy, all patients must be evaluated for both latent and active tuberculosis infection. Treatment is performed according to local guidelines for anti-tuberculostatic therapy [23]. PwMS who have not had chickenpox and who have not been vaccinated against Varicella-zoster virus (VZV) need to be tested for anti-VZV antibodies. Vaccination against VZV is required in seronegative patients. Several cases of opportunistic infections including cytomegalovirus, esophageal candidiasis, pyogenic granuloma, nocardiosis, and spirochetal gingivitis are reported on an anecdotal basis [23,24]. Listerial infections occur generally within 1 month of the treatment cycle [25]. Thus, dietary recommendations are provided and require the abstinence from certain foods during treatment with alemtuzumab and for the subsequent four weeks.

### 2.2. Cladribine

Cladribine (Mavenclad^®^, 2-chloro-2′-deoxyadenosine, Cd, Merck, The Netherlands) is a pro-drug that requires intracellular phosphorylation to become an active purine nucleoside analog. The active components interfere with DNA synthesis and repair, and ultimately lead to cell death [26]. Lymphocytes feature a higher deoxycytidine kinase (DCK) to 5′-nucleotidase activity ratio than other cells types. Thus, the intracellular metabolites Cd-ATP and Cd-AMP accumulate to high levels, which selectively depletes these cells [27,28].

Parenteral cladribine was first developed against hairy-cell leukemia. The oral formulation of cladribine was tested in clinical trials of pwMS and showed durable effects on inflammatory disease activity and clinical progression [29]. The EMA approved cladribine for the treatment of active MS in 2017. The dosage of cladribine tablets is 3.5 mg/kg over 2 years, with 1.75 mg/kg being administered each year [11]. The two annual treatment cycles encompasses two treatment periods, one at the start of the first month and the other starting in the second month. The dosage depends on body weight, and daily intake spans over four or five days in which a single 10 or 20 mg dose of cladribine tablets is taken. The second treatment cycle needs to be postponed in patients with slow recovery of lymphocytes (lymphopenia Grade ≥  2) at the start of year 2 until the absolute lymphocyte count has exceeded 800/µL. Oral bioavailability of cladribine varies between 37–55% [27]. The mean terminal half-life of cladribine in individuals with normal kidney function is 6–8 h.

Cladribine appears to modify the disease course in pwMS by depleting peripherally circulating B and T cells, and subsequently the pool of autoreactive subsets [30]. In a dose-finding study of patients with RRMS, reductions in T cells and their subsets were dose-dependent. In contrast, B and NK cell changes were similar irrespective of dose schedule [31]. These observations were made for a total dose of cladribine of 3.5 mg/kg vs. 5.25 mg/kg. In addition, cladribine treatment led to changes in the absolute cell count, relative distribution of many lymphocyte subsets and lowering of TH17 subsets [32]. The relationship of these observations to clinical effects remains to be elucidated.

Depletion kinetics after the first course of cladribine tablets were analysed from samples of three trials. The pattern was characterized by a decline of 48–55% reduction from baseline for CD4+ T cells. The nadir was at 13, 24 and 13 weeks after treatment initiation in each trial, respectively. CD4+ T cell counts then stabilized. Further analysis of CD4+ T cell subtypes showed that naïve CD4+ T and memory CD4+ T cells were maximally depleted at week 13 after initiation of the first cladribine tablets course [30]. The reduction was 63% and 51% from baseline, respectively. Central memory CD4+ T cell counts were maximally depleted at week 24, which was −63% from baseline, and the depletion continued at 48 weeks. This resulted in a decrease in the percentage of memory CD4+ T cells in the CD4+ T cell pool over time; the reduction at week 48 was a 9% reduction. Effector CD4+ T cell counts were maximally depleted at week 13 after initiation of cladribine tablets, which was −54% from baseline, and depletion was maintained at 48 weeks. However, the percentage of effector CD4+ T cells in the CD4+ T cell pool increased by 16% at week 48 [30].

The kinetics of CD8+ T cell depletion was similar to that of CD4+ T cells. Yet, depletion of CD8+ T cells was overall less pronounced, and recovery was faster. After the first course of cladribine tablets in the aforementioned three trials, the nadir for CD8+ T cell counts was observed at 48, 24 and 24 weeks after treatment initiation, respectively. The reduction from baseline ranged from 30–48%. Starting at the first dose of cladribine tablets, CD8+ T cell counts at no time decreased below reference values over a 240-week follow-up period [33].

Oral cladribine has a greater effect on B than T cells in peripheral blood. A rapid fall in B cell counts by 81–84% from baseline was observed, with the nadir at week 13 from treatment initiation [34]. B cell counts then return towards reference values, reaching levels about 30% below baseline by week 48. A similar pattern of depletion and repopulation occurs after the second annual treatment cycle [31]. Within the B cell population, magnitude and kinetics of depletion vary substantially. The ratio of DCK to 5′-NTase expression is particularly high in mature, memory and notably germinal center B cells. This is not the case for plasma cells. Depletion of class-switched and unswitched memory B cells is comparable to that with alemtuzumab.

Cladribine tablets also deplete various innate immune cells, although to a lesser extent than lymphocytes. Cell group studies include NK cells and monocytes. Reductions in neutrophils, platelets and erythrocytes are modest and mean levels remain within the reference range.

Lymphopenia is dose-dependent, and the nadir is at 4 months. Grade 3 lymphopenia (500–200 cells/µL) developed in around 25% of patients in the approved dosage, and grade 4 (<200 cells/µL) in less than 1% [35]. The rate of common infections is similar when comparing placebo- and cladribine-treated patients. The rate of herpes zoster infections was higher for the approved cladribine dosage than in the placebo group. There was a close relationship with lymphopenia. Subsequently, patients with grade 4 lymphopenia receive a prophylactic anti-herpetic treatment. Furthermore, the incidence of severe infections was generally higher among patients with lymphopenia [35]. Progressive multifocal leukoencephalopathy (PML) was not reported during an observational period of >8500 patient years in the MS indication. Importantly, PML cases have been observed with parenteral cladribine in lymphoma patients. Three cases of tuberculosis were reported during the clinical trials, of which one case was fatal [23]. Two cases of hepatitis B occurred; one patient died. Thus, clinical follow-up, standard laboratory tests, and screening for HIV infection, active tuberculosis, and hepatitis B/C are mandatory prior to treatment with cladribine [23].

### 2.3. Ocrelizumab

The concept of applying B cell-depleting therapies in MS has evolved predominantly on the assumption that antibodies autoreactive to a yet unknown antigen of the CNS are causal in the pathogenesis of the disease. Monoclonal antibodies against CD20 selectively deplete B cells. In this regard, immature and mature B cells are depleted but plasma cells and hematopoietic stem cells are spared due to their lack of CD20 expression. There is also a small subset of T cells expressing CD20 [36]. Rituximab was the first anti-CD20 antibody to be studied in MS trials. These early studies reported a rapid decline in the development of new CNS lesions in patients with RRMS [37,38]. Using ocrelizumab (Ocrevus^®^, Roche, Switzerland), the humanized successor, a substantial reduction in the frequency of clinical relapses and CNS lesion formation was corroborated in RRMS [39,40]. The approval of ocrelizumab in RRMS was based on two identical, randomized trials. These were double-blind, double-dummy trials comparing IV ocrelizumab to SC IFN-ß-1a. Ocrelizumab was also superior to IFN-ß-1a with respect to confirmed disability progression at 12 and 24 weeks. In addition, a placebo-controlled, phase-III trial in patients with primary progressive MS (PPMS) revealed a significant slowing for the accumulation of disability. This was most evident in younger pwMS and presence of MRI findings suggestive of ongoing inflammatory activity [41]. Thus, ocrelizumab was approved for both RRMS and early PPMS in 2018 by the EMA.

Re-administration of CD20 depleting agents is required on a regular basis and distinguishes them from alemtuzumab and cladribine in this aspect. Yet, ocrelizumab induces long-term depletion of memory B cells and the requirement for such frequent re-administration has been questioned [42]. Ocrelizumab is administered IV every 24 weeks at a maintenance dose of 600 mg. Within this interval, most patients are continuously depleted of peripheral blood B cells. Further research is required to unravel the effects of ocrelizumab in secondary lymphoid organs.

Both CD20+ B and T cells are rapidly and almost completely depleted two weeks after a single dose of ocrelizumab [43]. T cell counts are significantly reduced at 12 (−60%) and 24 weeks (−67%) from infusion, respectively. In patients with RRMS, ocrelizumab every 24 weeks for 96 weeks reduced counts of T cells, CD4+ T cells, and CD8+ T cells; the changes from baseline were −4%, −1%, and −9%, respectively at the end of the treatment period [34]. In patients with PPMS, changes from baseline in T cell, CD4+ T cell, CD8+ T cell counts were like those reported for placebo 120 weeks after the start of treatment. The changes were −4% vs. +3%, +1% vs. +6%, and −13% vs. −1%, respectively [44]. Data on long-term effects of B cell depletion in MS and, in particular, on changes in immunoglobulin levels, are eagerly awaited.

The anti-CD20 monoclonal antibodies rituximab and ocrelizumab are different from each other in certain aspects. Rituximab, which has not been studied in a pivotal phase-III trial, is used as off-label therapy in MS. This chimeric antibody acts predominantly via CDC. Ocrelizumab is more humanized, and its B cell-depleting effector mechanism is mediated more by ADCC [34]. A third anti-CD20 antibody is ofatumumab (Kesimpta^®^, Novartis, Switzerland), a fully human anti-CD20 antibody [45]. Ofatumumab was approved in 2020 by the US Food and Drug Administration (FDA) as once-monthly subcutaneous injection for the treatment of RRMS in adults. The approval by the EMA is expected in Q2 2021.

Patients taking ocrelizumab in the RRMS and PPMS trials experienced a higher incidence of infections [46]. Indeed, a systematic review of the literature revealed that infections were more likely in ocrelizumab-treated pwMS compared to IFN-β [47]. The infections were mostly related to herpes viruses and involved the respiratory tract.

## 3. The Host Immune Response to SARS-CoV-2 Infection

Over a short period of time, major insights into the host immunological response to SARS-CoV-2 were gained. Coronaviruses are enveloped, single-stranded RNA viruses that are susceptible to mutation and can infect humans via the respiratory system [48]. SARS-CoV-2 infection activates innate and adaptive immune responses. However, both uncontrolled inflammatory innate responses and impaired adaptive immune responses may have deleterious effects, both locally and systemically [49,50]. SARS-CoV-2 attaches to the host cell at the angiotensin-converting enzyme (ACE)-2 receptor [51]. SARS-CoV-2 specifically binds via the spike glycoprotein in the crown structure, which leads to alteration in the viral wall envelope. This virus–human cell interaction triggers the release of numerous immune mediators, including cytokines and chemokines aimed at destroying the virus [52]. In this regard, the levels of CXCL10, IL-6 and IL-10 in the blood have been shown to be associated with outcome [53].

Immune profiling studies have been instrumental in characterizing the spectrum of and the immune dysfunctions associated with SARS-CoV-2 infection. This includes evidence of SARS-CoV2 antigen-specific CD4+ and CD8+ T cells in acute COVID-19. Moreover, SARS-CoV-2-specific CD4+ and CD8+ T cell responses are detected in recovered patients ([54,55]. Several studies corroborated that lymphopenia occurs in COVID-19, which is associated with the extent of inflammation and disease severity [52]. Severe disease is characterized by significant dysregulation of the innate immune responses. There is an expansion in the proportion of both neutrophil and eosinophil populations, decreased expression of CD15 and CD16 on neutrophils (but not in eosinophils), lower frequencies of dendritic cells (both conventional and plasmacytoid dendritic cells), and a drastic decrease in the frequencies of both CD56brightCD16− and CD56dimCD16+ NK cells [56]. Patients with mild-to-moderate COVID-19 have activated CD4+ and CD8+ T cells, increased antibody-producing cells and follicular helper T cells, as well as IgM and IgG antibodies in blood prior to symptomatic recovery [57]. In patients with severe disease, peripheral T cells appear to be more activated based on the presence of an increased proportion of cytotoxic CD8+ T cells expressing CD38 or co-expressing CD38 and PD-1, compared to moderate disease and healthy controls [56]. There is expansion of plasmablasts, evidence of extrafollicular B cell activation, oligoclonal expansion of antibody clones within the overall B cell repertoire, and a strong SARS-CoV-2-specific antibody response in severely affected patients [56,58]. Hyperactivated T cells in severe disease also exhibit a trend toward cellular exhaustion. Pro-inflammatory responses such as increases in IL-6- or granulocyte-macrophage colony-stimulating factor (GM-CSF)-producing CD4+ T cells in the blood and decreases in immunoregulatory regulatory T cells (Treg) or γδ T cells are further observations [52].

Admittedly, some of the immune response characteristics observed in severe COVID-19 (e.g., decreased expression of CD16 on neutrophils, monocytes and immature granulocytes) bear resemblance to immune dysregulation observed in sepsis. Other observations (e.g., expansion of antibody-secreting plasmablasts and activated T cells) are more specific for an acute viral infection. Most importantly, SARS-CoV-2-specific CD4+ T cells and SARS-CoV-2-specific CD8+ T cells were associated with less severe disease. This leads to the assumption that T cells play a critical role in the control of active SARS-CoV-2 infection [59]. In contrast, the presence of neutralizing antibodies was not associated with disease severity.

## 4. Disease-Modifying Drugs and COVID-19: Restriction for Immune-Depleting Therapies?

Individualized effective disease control with attention to the risk of side-effects is the primary consideration in the decision making for DMD in MS [45]. DMDs alter the immune system in various ways, which may be of relevance for susceptibility and clearance of SARS-CoV-2 infection [60]. On the other hand, hyperinflammation is the leading cause of mortality in COVID-19, so the exact impact of different DMDs on the COVID-19 course is therefore eagerly awaited. Meanwhile, several national and international professional associations as well as patient organizations have issued practical guidance on limiting certain DMDs during the pandemic [61].

The Italian recommendation for the use of immunodepleting DMDs at the start of the COVID-19 pandemic was as follows [62]:

1. Temporarily delay (between 6 and 12 months depending on the DMD re-dosing of alemtuzumab, ocrelizumab and cladribine. This decision should be made according to individual factors such as disease severity and activity.
2. For anti-CD20 DMTs, it is recommended to delay the next dose even beyond 6 months if CD19+ and CD20+ lymphocyte counts are severely decreased at the time the next dose is due.

The motivation for a hierarchy is based on potential hazards related to immunosuppression or modulation of the host defense. Adaptive antiviral responses are driven in the acute phase mainly by T-cells, in particular CD8+ T and NK cells. Of note, the assessments were not uniform, the recommendations were slightly adapted over time, and are to be considered on an individual basis for clinical decision-making and patient counseling. However, there was consensus for restricting the initiation of lymphodepleting immunotherapies such as alemtuzumab, cladribine or antibodies targeting the CD20 antigen for the duration of the pandemic. For these therapies, an additional temporary delay between 6 and 12 months for re-dosing has been suggested. Despite different modes of action, these DMDs are characterized by short and repeated treatment courses with sustained treatment effects over the treatment free periods [34]. The temporary decrease or depletion of immune cells can last several months, and the subsequent loss of immune competence is a consideration on the one hand. On the other hand, humoral immune responses, which may be of relevance for viral clearance, may be attenuated and subsequently lead to a more severe and prolonged COVID-19 course. The concerns about increased vulnerability to infections for patients with immune-depleting therapies remain to be proven. Moreover, the viewpoint for a restricted use of pulsed immunotherapies during COVID-19 is not universal.

By looking at the immunological trajectories of the various outcomes for COVID-19 in the general population, antibody levels for both IgM and IgG were significantly lower in non-survivors, suggesting that the antibody response contributes to recovery [44]. A recent study corroborated a similar frequency, risk factors, and outcomes for COVID-19 in pwMS compared to the general population and regardless of DMD use [63]. An exception was treatment with CD20 depleting antibodies for a longer period, for which a higher risk of COVID-19 was found. Interestingly, in this study only 46% of the total cohort of pwMS with Polymerase Chain Reaction (PCR)-confirmed SARS-CoV-2 infection had serum antibodies formed approximately 3 months after symptom onset, shedding new light on the role of the humoral immune response in viral clearance.

## 5. Considerations for Vaccination in the Context of Immune-Depleting Therapies

Global vaccination against SARS-CoV-2 in combination with established primary prevention measures to reduce person-to-person transmission will be the critical tool to bring the pandemic under control [64]. Moreover, a vaccine raises legitimate hopes that these depleting therapies, which are categorized as higher efficacy DMDs and are therefore an indispensable tool in the armamentarium for controlling disease activity in MS, can be used more widely before the end of the pandemic. Yet, there are general considerations in this regard.

First, as shown in the clinical trials on the efficacy of these vaccines, immunization will not provide a complete protection. For instance, BNT162b2, a lipid nanoparticle–formulated, nucleoside-modified RNA vaccine, was 95% effective to prevent COVID-19 in persons 16 years of age and older [65]. The vaccine efficacy was 90 to 100% across subgroups stratified by age, sex, race, ethnicity, baseline body-mass index, and the presence of coexisting conditions. Another study evaluating an mRNA vaccine (mRNA-1273) demonstrated adequate protection for infection with risk reductions ranging from 90.9 to 94.4 percent for individuals at increased risk for severe infection (chronic lung disease; heart disease; severe obesity, BMI ≥ 40); diabetes (type 1, type 2, or gestational diabetes); liver disease; or human immunodeficiency virus infection) [66]. Despite this phase 3 evidence in a vulnerable population, the development of an adequate antibody response in pwMS needs to be evaluated. Evaluation of the response to vaccination and boostering may be required to maintain a favorable risk-benefit ratio.

Second, most pwMS are either already on immune depleting DMDs or are switching from non-depleting DMDs [65]. So far, there are no SARS-CoV-2 vaccine trials evaluating the efficacy while on certain DMDs approved for MS. In the VELOCE study, peripherally B-cell-depleted ocrelizumab recipients mounted attenuated humoral responses to clinically relevant vaccine antigens [67]. Among the more effective DMDs, the efficacy of vaccination has been tested for S1P receptor modulators, natalizumab and alemtuzumab. A systematic review revealed that most of the trials included only a small number of individuals and the results were not uniform [68]. Indeed, some studies reported a slower development of antibodies and lower rate of protection. In the context of MS, vaccination trials for cladribine have so far not been published. It therefore can be concluded that vaccination should preferably be completed before the start of the aforementioned DMDs.

Measures to confirm sufficient protection based on antibody titers is reasonable for patients vaccinated during DMD therapy. Well designed studies that establish the ideal timepoint for vaccination are warranted. For vaccination of pwMS in whom therapy with immunodepleting agents is already established, consideration may be given, and if clinically justifiable, to delay futher treatment cycles until after vaccination.

Third, functional hallmarks of antiviral immunity are observed in survivors of mild SARS-CoV-2 infection [69]. The adaptive immune response during COVID-19 is heterogeneous and the question of how well and how long the immune responses can protect the host from re-infection remains to be elucidated. For some viruses, the first infection can provide lifelong immunity, whereas there is evidence for short-lasting immunity from infection with seasonal coronaviruses [70]. Whether reinfections occur because of a scant antibody response after the first infection or whether genetic discordance of the SARS-CoV-2 strain is important is a key question [71].

Further research needs to be dedicated to a better understanding and assessment methods of persistent functionally relevant immunity to prevent treatment decisions based on the untrue presence of adaptive immunity against SARS-CoV-2.

Fourth, vaccinations in MS are generally considered safe. Live vaccines should be avoided during immunosuppressive therapy [72]. However, vaccines against SARS-Cov2 represent new types of vaccines. mRNA vaccines have already been mentioned and should be considered safe for people with autoimmune diseases, although autoimmunity due to the inflammatory response (e.g., type I interferons) cannot be 100% ruled out at this time [73]. Similarly, vector vaccines represent a novel type of vaccination. Here, components of the vaccinating viral strain are implanted into a harmless virus (vector). As with mRNA vaccines, there is no replication of the virus [74].

For both vaccination strategies, and especially in the pandemic, experience is limited and treating physicians must be vigilant to new reports and subgroup analyses. Immunodepleting therapies pose a risk for infection. Avoiding such therapy because of the risk of infection and infection with SARS-Cov2 are risk factors for worse outcome in pwMS; vaccination may be a solution to both risks.

## 6. Conclusions

Neurologists treating people with immunosuppressive and immunomodulatory agents must balance the risk of SARS-CoV-2 infection and its potentially life-threatening course against a possible progression of the underlying condition. The mode of action and hitherto real world evidence implies that not all DMDs are associated with equal risk of severe SARS-Cov2 infection in pwMS [59]. As the end of the pandemic is not yet in sight, vaccination against the virus may be an elegant strategy to overcome the potential hazards associated with immune-depleting agents and the subsequent restricted use of these therapies. Moreover, the viewpoint on the acceptable risk with regard to loss of disease control vs. protection against COVID-19 is expected to change in the further course of the pandemic. At the current stage, many questions regarding innate and adaptive immune responses against SARS-CoV-2 and persistent protection remain to be elucidated.

## Figures and Tables

**Table 1:** Overview of pulsed immunodepleting therapies approved for the treatment of MS and their extent of lymphopenia, adapted from [7].

	Alemtuzumab (Lemtrada^®^)	Cladribine (Mavenclad^®^)	Ocrelizumab (Ocrevus^®^)
**Indication**	RRMS	RRMS	RRMS/PPMS
**Route of administration**	i.v.	oral	i.v.
**Dosing/Interval**	A total of 8 treatment days separated into two annual pulsed treatment periods: 5 × 12 mg (1st year) and 3 × 12 mg (2nd year)	Maximum 20 treatment days separated into two annual pulsed treatment periods: total of 3.5 mg/kg body weight (given as 1.75 mg/kg per year for each treatment period)	Induction with 300 mg at day 0 and 14 each, followed by maintenance dose of 600 mg every six months
**Degree of lymphophenia #**	Grade 3 and 4: 99.9%	Grade 3: 25.6%, Grade 4: 0.7%	Grade 1 and 2: majority, Grade 3: 1%
**Lymphocyte recovery #**	Total lymphocytes: 6 At 6 and 12 months in 40 and 80% of patients after each treatment cycle, respectively.	Recovery of lymphocytes at the end of each treatment year: 86%	After 2.5 years: 90% of patients had recovered CD19+ B cells

Legends: RRMS relapsing-remitting MS, PPMS primary progressive MS, i.v. intravenous. Rituximab (i.v., induction and maintenance dose is variable, mostly 500 mg every 6 months) is not listed due to off-label use. Grading of lymphopenia: Grade 1 (mild) absolute lymphocyte count (ALC) 800/µL to lower limit of normal to, Grade 2 (moderate) ALC 500–800/µL, Grade 3 (severe lymphopenia (severe)) 200–500/µL, Grade 4 < 200/µL. # in Phase 3 trials or subanalyses.
